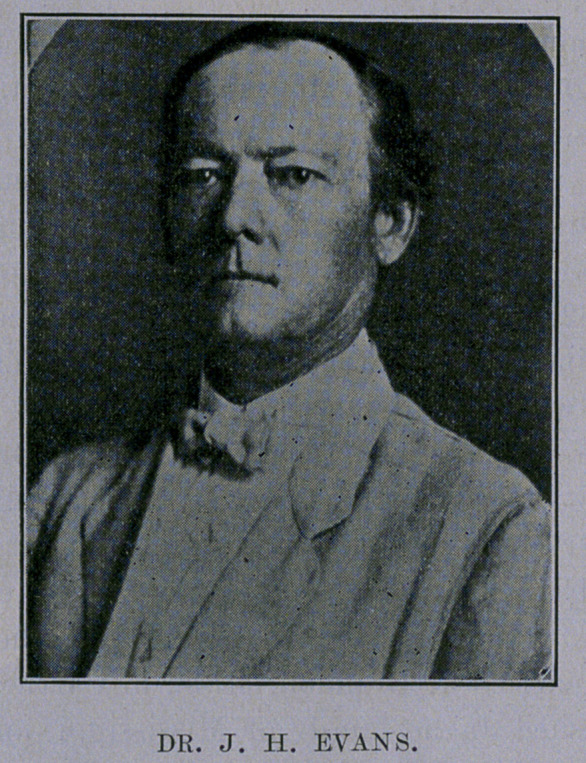# The Passing of Dr. J. H. Evans

**Published:** 1914-04

**Authors:** 


### The Passing of Dr. J. H. Evans.

On February 24th the sad news was heralded of the death of Dr. J. H. Evans, of Palestine, Texas. It came as a shock and surprise to his many friends both within and without the profession. His death came suddenly and while he was in full vigor of health and engaged in attending his practice. He was seated

DR. J. H. EVANS.

in his office talking with one of his professional friends when he was stricken, dying in a few minutes.

Dr. Evans was born in Kountz, Hardin county, Texas, fifty-eight years ago. He attended the public schools of his county, and received his medical education from Tulane University, New Orleans, and graduated in 1884. He began practice at Alto, where he remained for eight years, removing to Palestine, where he lived up to the time of his death. His work as a surgeon had been recognized throughout the State. He identified himself with every movement for the uplift of humanity. He was an ex-president of the Anderson County Medical Society and at the time of his death was president of the State Board of Medical Examiners. He was appointed medical examiner in 1901 and served longer in that capacity than any other member of the board. The fact that he was appointed by three different Governors of the State is a splendid testimony to his worth, his ability as a physician, and his upright, honest, courageous standing. Having been associated with him for two years on the board the writer can testify to his splendid character, his kindness of heart, his keen sense of justice, and fearlessness in the discharge of his duties. There is no higher praise that can be bestowed upon a man than to say that he is honest, and this could truthfully be said of Dr. Evans. He was brave in the face of adversity, uncomplaining, and extremely thoughtful of the welfare of others. He was a good friend, physician, father, and citizen, and when this is said of him there remains little more to be added. Those of the profession who were acquainted with him know that his ideals were of the highest and that he worked hard to keep abreast with the profession.

His death, coming as it did, suddenly, while causing a profound shock to those who loved him, should be considered as the canceling of a debt, that must be paid, in a manner that brought to him no suffering, prolonged illness or helplessness of old age. It should be looked upon as a surcease from toil and the peaceful rest of a beautiful life amidst the surroundings of beloved friends and family and with the full esteem of his colleagues.

He has "crossed the bar" and need not fear "meeting his Pilot face to face."

Surviving him are his wife, six children and one grandchild, viz: Mrs. T. M. Norsworthy and daughter, Sylvia Orlone Norsworthy, of Houston; Elliott J. Evans, Miss Orlone Evans, J. H. Evans, Jr., Will R. Evans, and Culberson Evans, all of Palestine, Texas.

## Figures and Tables

**Figure f1:**